# Epigenetic Changes Occurring in Plant Inbreeding

**DOI:** 10.3390/ijms24065407

**Published:** 2023-03-12

**Authors:** Magdalena Achrem, Edyta Stępień, Anna Kalinka

**Affiliations:** 1 Institute of Biology, University of Szczecin, 71-415 Szczecin, Poland; anna.kalinka@usz.edu.pl; 2 Institute of Marine and Environmental Sciences, University of Szczecin, 71-415 Szczecin, Poland; edyta.stepien@usz.edu.pl

**Keywords:** inbreeding, epigenetic mechanisms, inbreeding depression, epiRIL

## Abstract

Inbreeding is the crossing of closely related individuals in nature or a plantation or self-pollinating plants, which produces plants with high homozygosity. This process can reduce genetic diversity in the offspring and decrease heterozygosity, whereas inbred depression (ID) can often reduce viability. Inbred depression is common in plants and animals and has played a significant role in evolution. In the review, we aim to show that inbreeding can, through the action of epigenetic mechanisms, affect gene expression, resulting in changes in the metabolism and phenotype of organisms. This is particularly important in plant breeding because epigenetic profiles can be linked to the deterioration or improvement of agriculturally important characteristics.

## 1. Introduction

The variability of characteristics observed in natural and experimental populations of plants, which are important for agriculture and evolution (e.g., yield, flowering time, resistance to pests and drought), is usually explained by the occurrence of the DNA sequence polymorphisms and the interaction of environmental factors. Currently, it is believed that the heredity of complex characteristics is not only based on the transmission of the stable DNA sequence variants to progeny [1], but it is also linked to the transmission of specific patterns of chromatin modifications, i.e., epigenetic modifications from one generation to the next [2]. Examples include specific DNA methylation patterns that are reflected in the phenotype, e.g., in traits such as the flower shape and size or fruit pigmentation [3,4,5].

Inbreeding can lead to a reduced genetic diversity in the progeny as heterozygosity is limited, reduced and can lead to reduced viability associated with inbreeding depression. Despite the reduced genetic diversity resulting in the lower evolutionary potential, self-pollinated plant lineages are widespread, even in invasive species [6]; moreover, inbreeding can positively influence vigour and fertility under new environmental conditions with a few cospecies. This shows that adaptation to certain environmental conditions can also be associated with a reduction in genetic variation, when a particular phenotype is better adapted to certain environmental conditions, and this is maintained through inbreeding [7]. Epigenetic mechanisms play an additional role here as they are integral to the regulation of many processes in the genome [8,9]. 

Epigenetics encompasses numerous mechanisms that act at the transcriptional level by altering the chromatin structure, but also post-transcriptionally, regulating the gene expression activity [10]. The chromatin structure affects the accessibility of DNA to transcription factors and is regulated by epigenetic mechanisms, such as the DNA methylation, the histone variant exchange, post-translational histone modifications, chromatin remodelling and the interaction of non-coding RNAs (ncRNAs) [11,12]. 

The development of new technologies, especially state-of-the-art sequencing technologies, enables further understanding of epigenetic mechanisms. The research over the last 20 years has shown how important epigenetic mechanisms are for the adaptation of plants to new or varying environmental conditions, as well as in plant breeding. Epigenetic variation, through the so-called epibreeding, can be used to optimize plant breeding and improve crop resilience [13]. A valuable source of phenotypic variability are epialleles, which do not occur in nature very frequently. Epigenomic analyses (map-based genetic cloning, genome wide methylome analysis) are necessary to identify epialleles, enabling the identification of epigenetic changes and their association with the change in the phenotype [14]. For the same purpose, recombinant inbred lines (RIL) from germplasm with high epigenetic diversity are applied [15]. Another source of natural epigenetic variation are stress-induced changes [16,17,18]. One of the strategies used to obtain epigenetic variation is a targeted epigenetic modification, which uses systems such as TALE, ZF proteins or CRISPR-Cas9 to edit the epigenome, enabling changes in the gene expression, e.g., to improve agricultural properties [19,20,21]. In order to reduce the need to use plant transformation systems, virus-induced transcriponal gene silencing (VITGS) can be used to obtain epigenetic modifications in plants [22]. Nevertheless, the changes obtained are not always stable and hereditary [23]. Epigenetic recombinant inbred lines (epiRIL)—created as a result of wild plant crossing with hypomethylated mutant—are also widely used in epibreeding (*met 1* and/or *ddm1*). They are characterized by a variety of features, such as plant height, flowering time, yield, plant stress tolerance [21,24,25]. The work using epiRIL has hitherto focused on Arabidopsis, but the potential of this strategy is very extensive. The development of epiRIL in Arabidopsis also allowed the mapping of epigenetic loci of quantitative traits (epiQTL) [26], which enables the analysis of the role of the epigenetic variability in the regulation of agricultural properties. Due to the lack of DNA methylation mutants in crop plants [27], pharmacological strategies with DNA MTase inhibitors or the MSH1 system are used, which lead to changes in DNA methylation, affecting the yield and resistance of plants [28,29,30,31,32]. An example of such “epimutagenesis” is the development of the *Oryza sativa* line, resistant to *Xanthomonas oryzae*, after having been treated with 5-azacytidine [28]. A repeated selection of isogenic lines makes it possible to obtain stable epigenotypes and to identify epigenetic elements affecting agronomic properties, which can be used in epibreeding [14]. Epigenomic studies can be the basis to create phenotype prediction models, e.g., epiRIL was used to obtain predictive models of plant height [27], or to predict the level of the gene expression [33]. In order to reveal new epialleles associated with adaptation to new environmental conditions, epihybrids (created as a result of crossing epigenetically different inbred lines) can be used, which often exhibit heterosis [34]. Epigenetic changes could become a biomarker that will indicate a beneficial or pernicious phenotype, conditioned by epiallele data, and will allow us to learn about the molecular regulation of agronomic properties, which can be used in plant breeding [35].

## 2. Epigenetic Mechanisms

Cytosine methylation in DNA is a conservative chromatin modification that involves the attachment of a methyl group (CH3) to the 5-carbon position of cytosine (5 mC—5-methylcytosine). The methylation of cytosine plays a key role in regulating gene expression and ensuring the integrity of the genome by, e.g., the silencing of mobile elements. It is also directly involved in genomic imprinting and the transgenerational epigenetic memory [36,37]. In plants, DNA methylation occurs in three sequence contexts that are initiated and maintained by separate enzymatic pathways and correlate strongly with the methylation of lysine 9 in histone H3 (H3K9me) [38]. The most common is symmetric methylation at the CG dinucleotide (CpG), catalysed and maintained by the DNA Methyltransferase 1 (MET1), which corresponds to DNMT1 (DNA methyltransferase 1) in animals [39,40]. Cytosine methylation can still be symmetrical in the context of CHG and asymmetrical in CHH (where H = A, C or T) (reviewed in [41]). Cytosine methylation plays an important role in silencing the transcriptional-level expression (TGS) of transposable elements and repetitive sequences [42,43,44], which ensures that the stability of the plant genome is maintained. DNA methylation also usually leads to the inactivation or reduction of the gene transcription, especially when it affects the promoter region. In this case, it prevents the recruitment of transcription activators and allows the binding of repressors for this process. In contrast, the gene body methylation (gbM) is not always associated with silencing [45,46]. 

Chromatin contains numerous post-translational histone modifications (PTMs) that form a histone code, enabling the recruitment of specific proteins that determine the transcriptional potential of a particular gene [47]. PTMs include methylation, acetylation, ubiquitination, SUMOylation, phosphorylation, carbonylation and glycosylation [48,49]. These modifications can undergo dynamic changes due to the activity of proteins and histone-modifying complexes involved in the attachment, maintenance or removal of these markers. The most important PTMs include histone acetylation and methylation. In general, histone acetylation leads to a reduction in electrostatic interactions between histones and DNA, allowing better access to DNA [11]. Histone methylation can have both repressive (H3K9me2/3, H3K27me3, H4R3me2—symmetric) and activating (H3K4me3, H3K36me2/3, H4R3me2—asymmetric) effects on transcription [50]. The presence or absence of post-translational modifications of histones leads to a change in the chromatin structure and alters interactions with the corresponding “histone decoding proteins”, thereby altering the gene expression in that region [51,52]. Histones can also be exchanged for histone variants, which alters the stability of nucleosomes and directly affects the availability of DNA to various proteins, including transcription factors. This alters the activity of genes in a particular chromatin region, enabling or inhibiting transcription [48,53]. For example, histone variants H2A.Z or H3.3 are characteristic of euchromatin regions, especially at gene loci with moderate to high levels of transcription [54]. 

One of the most important types of regulatory non-coding RNA molecules are small RNAs (sRNAs), which are between 8 and 30 nt long [55]. Based on their biogenesis and structure, we can divide them into two main classes: microRNAs (miRNAs) and endogenous short interfering RNAs (siRNAs). The control of the gene expression by sRNAs occurs mainly at the post-transcriptional level. Gene silencing can occur through the mRNA degradation or repression of transcript translation [56] (reviewed in [57]). 

The regulation of the gene expression involving siRNA and miRNA molecules occurs not only at the post-transcriptional level, but also at the transcriptional level, leading to the transcriptional gene activation (TGA) and transcriptional gene silencing (TGA) [58,59].

The interactions between DNA methylation, histone PTMs and the mechanism of action of ncRNAs allow plants to control the gene expression at multiple levels. One example is the regulation of expression of many miRNA genes by DNA methylation and histone PTM. Depending on histone modifications, miRNA genes can be transcriptionally active or inactive. The methylation of these genes within the CpG island inhibits the expression of the respective miRNA. Furthermore, it was found that hydroxymethylated cytosines (resulting from active demethylation) can act as transcriptional enhancers of miRNAs [60]. 

In mammals, the formation of tissues and organs takes place mainly during embryonic development, whereas in plants, new tissues and organs are formed from meristematic cells throughout their whole lives. Therefore, the post-embryonic development in plants is more strongly influenced by environmental factors, which leads to a high degree of phenotypic plasticity. Furthermore, due to their settled lifestyle, plants have to adapt to changing environmental conditions that are often unfavourable for them. The rapid response of epigenetic mechanisms can facilitate the adaptation of the gene expression. Epigenetic regulatory mechanisms can facilitate metastable changes in the gene activity and fine-tune gene expression patterns, enabling plants to survive and successfully reproduce in variable environments, even under inbreeding [25,61].

## 3. Inbreeding and Inbreeding Depression

Inbreeding (kin-crossing) is the process of mating between individuals that are related to each other. It also occurs during self-pollination [62].

In large natural populations with a high genetic differentiation, crossing between unrelated individuals occurs most frequently. In small and isolated populations, inbreeding is often observed, with mating individuals that have greater genetic similarity than individuals crossing randomly in a large population [63]. If the rate of inbreeding increases rapidly in such populations, there may be a reduction in the adaptation of the progeny, which is known as inbreeding depression [64]. This can be associated with reduced germination, slower growth and reduced height or fertility compared to non-inbred plants [65]. Inbreeding increases homozygosity in the offspring generation, leading to a reduction in the genetic variation in the population and an increase in the expression of recessive deleterious alleles [62,66]. This often results in offspring that are weaker than the parents, which can reduce the plant’s chances of survival. The effect of inbreeding on the adaptation of an individual is referred to as the coefficient of inbreeding f (or: CI, CoI), which indicates the probability that two alleles at a randomly selected locus are identical because of common descent [67]. 

Hypotheses explaining the negative effects of inbreeding and the development of inbreeding depression are based on dominance and overdominance theories, which explain the greater adaptation of heterozygotes. According to the associative overdominance hypothesis, alleles that increase the adaptation of individuals are generally dominant, while recessive alleles are considered unfavourable. According to this theory, inbreeding increases the degree of homozygosity, resulting in inbreeding depression as a result of the increased expression of rare, unfavourable (deleterious) recessive alleles or negative epistatic interactions between homozygous loci [4]. The second theory is the functional overdominance of heterozygotes (functional overdominance hypothesis), according to which mere heterozygosity at multiple loci increases the adaptation of individuals, which is limited in the case of inbreeding [68].

In diploid organisms, self-pollination causes heterozygosity to be halved in each generation, although this decrease is slower in polyploid plants [69,70]. Inbreeding depression is expected to be lower in autotetraploid species compared to diploid species. This is due to a lower rate of progression to homozygosity [71] and better masking of deleterious mutations in a single copy of the gene [72]. However, the results of studies on inbreeding depression in both autotetraploid wild-type plants [73,74] and in cultivated plants [75,76] often show a higher level of inbreeding depression than expected, which is sometimes comparable to that observed in diploids [74,77]. 

The phenomenon of inbreeding depression is evident in the phenotype of affected plants and can relate to the plant size, flower size, germination capacity, seed yield or stress tolerance [78]. Inbreeding depression also depends on the life cycle of the plant [73,74,79,80]. The analysis of the phenotypic changes resulting from inbreeding depression can help to identify the genes involved, e.g., using a genome-wide association study (GWAS) [81]. This provides information about which traits and/or cellular processes may be altered in inbreeding depression.

As the rate of inbreeding increases, so does inbreeding depression, which can be exacerbated by environmental stress. Inbreeding in plants can result in lower resistance to biotic and abiotic stresses [82,83]. For instance, inbreeding may cause plants to be more susceptible to natural enemies [84,85]. Inbreeding can reduce the expression of genes whose products contribute to the formation of defence compounds [86,87], phytohormones [88] and metabolites [86,89] that are critical for defence signalling [90]. These changes in inbred plants may contribute to increased nutritional losses, leading to inbreeding depression in the presence of herbivores [89]. One example is *Datura stramonium*, in which inbreeding reduced the plant defence ability, leading to a 4% increase in the damage caused by herbivores and an 8% increase in viral infections [84]. Similarly, the research by Schrieber et al. [91] showed that inbreeding in *Silene latifolia* reduced the amount of metabolic compounds that mediate the plant resistance and deter herbivores. Previous studies revealed that inbreeding leads to a reduction in phenolic compounds that provide defence in *Vincetoxicum hirundinaria* [92] and *Solanum carolinense* [89].

In plants, inbreeding depression can be increased by factors such as disease, unfavourable field conditions or competition [93,94,95,96,97,98]. However, there is work that does not confirm this [82] and evinced lower inbreeding depression under stress [99]. Water stress had no effect on the level of inbreeding depression in *Raphanus sativum* [100]. Reducing nutrient stress in *Schiedea* by applying fertiliser resulted in the increased ID [95]. It was also found that the levels of ID can increase or decrease depending on the stressor [101,102,103,104]. In *Cucurbita pepo* ssp. *texana*, for example, nutritional stress increased ID, but the effects on the phenotype differed significantly during the four-year study [105]. In *Clarkia tembloriensis*, greater inbreeding depression was observed in the greenhouse than under field conditions [106]. According to Rehling et al. [107], the level of ID may be low in the case of stresses, to which plant populations were exposed for a long time, as deleterious alleles specific to the environment may have already been eliminated.

The effect of inbreeding on the occurrence of inbreeding depression also depends on the population size. In natural, large populations with inbreeding, a process of the reduction of unfavourable alleles, called purging, and genetic rescue is often observed [108]. The slightly unfavourable mutations that do occur are influenced by genetic selection, which increases the selection against them, resulting in their lower frequency. A different situation arises in small populations where deleterious mutations may accumulate due to the effect of genetic drift that prevents the selection against negative alleles [108]. 

Reduced flower size, along with the pollen and odour production, were observed in inbred lines of *Solanum carolinense* [109]. Similarly, the inbred lines of *Silene latifolia* had smaller flowers with a different spatial arrangement and reduced the odour production, which is necessary to attract moths (*Hadena bicruris*) involved in the reproduction of these plants [91]. This shows that inbreeding can contribute to the population decline, as the flowers are less attractive to animals. 

It is important to remember that inbreeding can also bring benefits. Humans use inbreeding to improve desirable traits, but in this case the individuals crossed must be selected very carefully. In raising animals and growing plants, inbreeding allows the accumulation of valuable alleles and their fixation in the population. Inbreeding reveals recessive genes. However, the frequency of unfavourable alleles is usually low and kin mating allows their identification and removal from the population. Inbred lines also provide a component for crossing to induce heterosis among them [110,111]. The more diverse the inbred lines are, the greater the heterosis effect that can result from masking of mildly deleterious mutations is. However, high diversity between crossed inbred lines can lead to hybrid depression [112].

## 4. Inbreeding and Epigenetics

**Figure 1:**
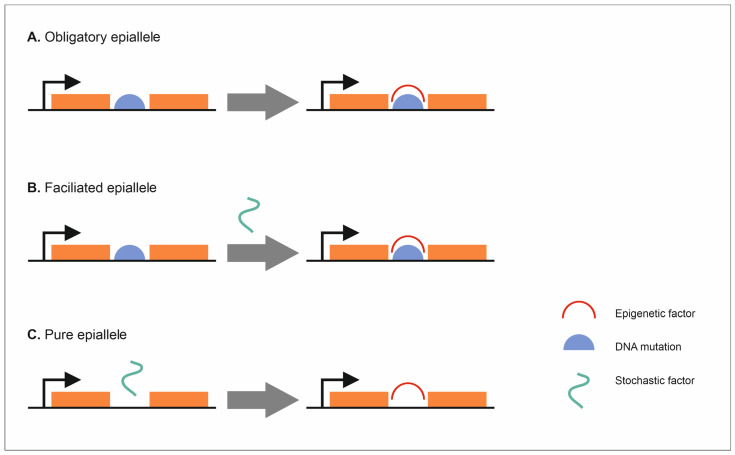
Types of epialleles. (**A**) Obligatory epialleles. (**B**) Facilitated epialleles. (**C**) Pure epialleles.

Inbreeding is associated with many genetic and epigenetic changes. One epigenetic mechanism associated with changes in the gene expression during inbreeding is the DNA methylation, as demonstrated by studies on inbred lines of maize [113,114], *A. thaliana* [25,61], and the analyses of the differences between the inbred parental generation and F1 hybrids [4,34,115]. For example, the analysis of maize inbred lines showed differences in the DNA methylation patterns between individuals, mainly due to changes in CHG or CG/CHG methylation. It was also found that, among DMFs (differentially methylation fragments), up to 12% were inherited for at least six generations [116]. The best example of heritable epigenetic changes are epialleles, i.e., different forms of the same gene that differ in the way the sequence is methylated and are inherited mitotically and/or meiotically. They can occur spontaneously or be induced and lead to altered gene expression and phenotypic variation [116,117,118]. Epialleles may be important for the occurrence of heterosis, inbreeding depression, genetic incompatibility or plant response to stress [119,120,121]. It is very important to know how epialleles are formed and inherited [122] as they can influence the phenotypic variation in traits, such as plant height, flowering rate, resistance to diseases, root length or total yield [1,123,124,125]. Richards [126] distinguished three types of epialleles based on their correlation with genetic variation. They range from completely dependent, obligate epialleles, through partially dependent, facilitated epialleles, down to independent, pure epialleles (Figure 1). The first type is closely associated with a genetic alteration, e.g., TE insertion. The hypermethylation of certain retrotransposons can cause the DNA methylation of neighbouring regions, leading to the formation of obligate epialleles [113,114] that have high stability and heredity. The second type of epialleles are those associated with an appropriate genetic variant, but not fully dependent on it. The last recognized type of epialleles is not related to genome variability [113]. However, the last two types of epialleles can be less stable than obligate alleles [114] (Figure 1). 

The first known epiallele is *clark-kent* (clk) in *Arabidopsis*, which is an epiallele of the *SUPERMAN (SUP)* gene. DNA hypermethylation in the body of the *SUP* gene was associated with the abnormal development of floral whorls [127,128], but this modification was not stable. In *Linaria vulgaris*, a stable epimutation was observed, which was associated with the hypermethylation of the *Lcyc* gene promoter, resulting in a different flower symmetry (radial symmetry) compared to wild-type plants (bilateral symmetry) [3]. In tomatoes, an epiallele of the *Cnr* gene (*COLORLESS NON-RIPENING*) carrying a hypermethylated promoter was observed [129]. The *Cnr* gene encodes an SBP box transcription factor (TF) and the hypermethylation of the promoter causes the fruit to fail to ripen. This epiallele is stably inherited; the *Cnr* epimutation was shown to rarely reverse [130], and is also associated with the phenotypic variation in traits such as the plant height, root length and resistance to diseases [1,28,124,125,131].

As mentioned above, many differences in the cytosine methylation between individuals or species correlate with genetic variation [114,132], but the epigenetic variation also arises in a gene-independent manner. An example is the stress-induced high diversity of DNA methylation patterns in apomictic dandelion plants with a low genetic variation [133]. The analyses of maize inbred lines also indicated evidence of the naturally occurring epigenetic variation in these plants that did not correlate with the genetic variation but resulted in phenotypic changes [113,114,116]. 

For inbred plants, phenotypic plasticity is very important to adapt to new and/or unfavourable environmental conditions. In plants with low genetic diversity, it is epigenetic changes that can influence the phenotypic plasticity and adaptability to new environmental conditions [25,61]. Phenotypic plasticity is defined as the ability of a genotype/epigenotype to produce several alternative phenotypes, depending on environmental conditions [134]. It can involve morphological and physiological changes, which are often associated with higher performance, e.g., of agriculturally important traits [135]. Epigenetic changes not only influence the silencing of ‘redundant’ genes in a given environment, but also increase the diversity of the gene expression between genome-identical individuals [61,136]. It is also important to note that an epigenetically inherited phenotype, in contrast to phenotypic changes that are due to mutations, can be visible in several individuals at the same time [137]. 

The contribution of DNA methylation to the phenotypic plasticity can be investigated by using the chemical inhibitors of DNA methyltransferases, such as 5-azacytidine [138] or zebularine [139], to demethylate DNA [140,141,142]. An example is the analysis of inbred lines of the perennial plant *Scambiosa columbaria*, which have higher levels of DNA methylation, which increases inbreeding depression in these plants. In contrast, the treatment of *S. columbaria* seedlings with 5-azacytidine leads to the elimination of negative inbreeding-related traits, such as fewer leaves or low photosynthetic efficiency [4]. Another example is the DNA demethylation in inbred lines of *Polygonum persicaria*, which illustrates the role of the DNA methylation in the plant adaptation to changing environmental conditions, in this case drought, irrespective of the genetic variation [142]. 

The inheritance of drought-induced phenotypic changes in successive generations of 12 *P. persicaria* inbred lines was also investigated. Parental plants exposed to drought and reared under optimal conditions were analysed. Some of the progenies of these plants were then exposed to zebularine, and subsequently all plants (from the control and zebularine-treated samples) were moved to dry soil. The progeny of plants grown under drought conditions and not treated with zebularine had an expanded root system, which made their biomass greater than that of the progeny of plants not exposed to drought. In contrast, zebularine-induced demethylation resulted in the disappearance of the drought-adapted developmental effects but had no effect on the phenotype of the progeny of the parental plants not exposed to drought. This indicates that DNA methylation mediates environmentally-induced phenotypic changes, leading to the improved adaptation and inheritance of these changes to the progeny [142]. Similar findings where demethylation removed adaptations in progeny were reported for salt stress in *A. thaliana* [143] or exposure to herbivores in *Mimulus guttatus* [141]. Furthermore, the treatment of inbred lines of *A. thaliana* with 5-azacytidine showed that demethylation significantly reduced the growth and condition of these plants and delayed flowering. It was also found that the response to demethylation was variable and did not correlate with genetic similarity between the inbred lines tested [144]. In contrast, studies with inbred lines of *Antirrhinum majus* showed that the global demethylation of the genome in these lines correlated with stem elongation in response to shading. This confirms that epigenetic changes are associated with phenotypic plasticity in plants [145]. 

The link between inbreeding and epigenetic mechanisms is well illustrated by the study of Han et al. [5] regarding maize. They found that inbreeding results in the hypermethylation of thousands of genomic regions at TCP-binding sites (TBS), which is accompanied by high di- or trimethylation of histone H3 (H3K9me2, H3K27me2 and H3K27me3). This high level of DNA methylation near TBS motifs in the promoters resulted in the reduced recruitment of the transcription factors ZmTCP (*Zea mays* TCP) and thus, a lower gene expression. The transcription factor TCP (Teosinte branched1/cycloidea/proliferating cell factor) controls plant development, as the target genes of the TCP transcription factor are involved in photosynthesis, energy metabolism and ribosome biosynthesis. Therefore, the reduced expression of these genes leads to reduced plant vigour. Moreover, the methylation of the CHH motif in TBS can influence inbreeding depression by modulating the diurnal clock (*ZmCCA1b* gene—maize CIRCADIAN-CLOCK ASSOCIATED1 (*CCA1*) homologs) and related genes involved in, e.g., the photosynthesis and starch metabolism, among others [5]. This suggests that inbreeding affects the regulation of TCP factor target genes involved in maize growth [5]. Another example shows that inbreeding can cause changes in the methylation of DNA and the expression of genes affected by TEs. Namely, the reduced expression of the metastable locus *red1* (*r1*) and the activity of the *mutator* (*Mu*) transposon in maize were associated with a higher methylation of DNA during inbreeding. In addition, the hypermethylation of TEs was observed during inbreeding, a process preventing expansion of TEs; however, hypermethylation interacted with neighbouring genes and affected their expression [5].

Another type of example is provided by the studies with the Chinese cabbage (*Brassica rapa* ssp. *pekinensis*). Inbred lines show large phenotypic changes compared to wild-type plants, although there are no genetic differences between them. This suggests that epigenetic mechanisms must be involved in the phenotypic changes. The DNA methylation level of the genome of inbred lines is significantly reduced and alters the expression of many genes that influence the occurrence of traits typical of inbreeding depression [146].

DNA methylation changes can additionally be generated using a variety of techniques. For example, DNA methylation patterns in crop plants can be influenced by using the *MSH1* (*MutS Homolog1*) gene suppression approach, using the RNAi mechanism [30,31,32]. In inbred lines of *Sorghum bicolor*, it is revealed that the epigenetic variability, induced by the MSH1 system, influenced plant yields and resistance. Regardless of whether selection was used or not, an increase in yield was observed, as in the case of normal and modified (low nitrogen content) field conditions. The MSH1 system can therefore be used in the design of appropriate breeding strategies by generating epigenetic changes [32].

Other studies of inbred maize lines show that DNA methylation is not always correlated with the modification of histones. For example, we observed the level of lysine 27 trimethylation of histone H3 (H3K27me3) varied among tissues of developing plants, which reveals the role of this modification in regulating the gene expression during plant development. In contrast, no such variation in H3K27me3 was observed between maize inbred lines [147,148] and for *Brassica rapa* L. [149]. In contrast, DNA methylation levels are more variable between inbred lines [147,148].

It has also been found that random crossing in a population of inbred lines can restore vigour and viability through heterosis. It turns out that epigenetic changes have a large impact on heterosis, especially through the reduction in the level of the DNA methylation; the research results indicate a high vigour of hybrids obtained as a result of crossing inbred lines, resulting from differences in gene expression. The analysis of the gene expression between inbred lines, used as parental components and progeny hybrids (6-day-old maize embryos), revealed differences in expression in 8.9–15.3% of genes [150]. Significant differences in the gene expression were also shown by the F1 progeny of inbred B73 and Mo17 maize lines, relative to their parents, both at the seedling stages and in the embryonic tissue [151]. The observed changes in the gene expression may lead to hybrid phenotypes with better or worse characteristics compared to the parental phenotypes [152,153,154,155]. What is more, for some traits, a correlation was found between the number of genes with different expression and the degree of heterosis [156,157]. Changes in gene expression between inbred lines and resulting hybrids are also caused by sRNA or histone modifications [158,159,160,161]. For example, Seifert et al. [158,159] assessed the sRNA variation in inbred and hybrid lines. They linked them to grain yield heterosis in the hybrid lines. The comparison of epigenetic changes in inbred lines and hybrids allows us to understand the potential role of these epigenetic mechanisms in heterosis [35].

## 5. Epigenetic Recombinant Inbred Lines 

**Figure 2:**
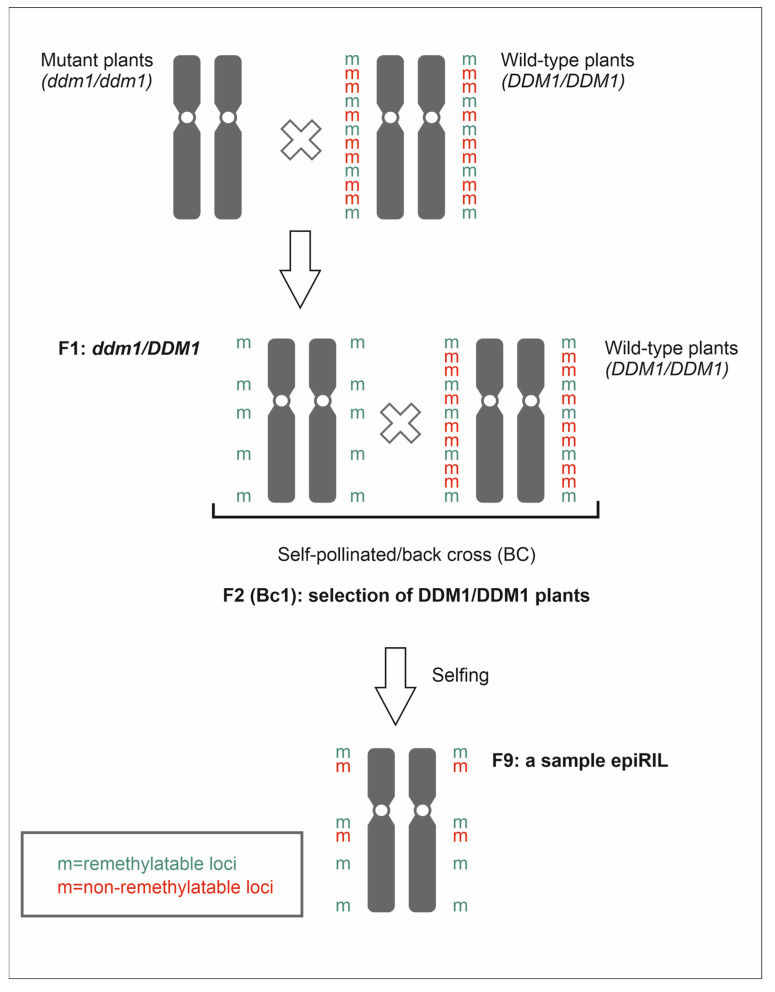
Scheme of epiRIL formation.

Natural epialleles are rare—therefore, populations of inbred lines, called epigenetic recombinant inbred lines, were developed to explore and understand the contribution of epialleles to transgenerational inheritance, the activation of transposable elements and phenotypic changes. EpiRILs were generated by crossing *A. thaliana* wild-type plants (WT) (ecotype; Columbia-0, Col-0) with met1 or ddm1 mutant lines. The resulting progeny were repeatedly self-pollinated until the obtaining of epiRILs that were homozygous in terms of a specific DNA methylation pattern [24,162] (Figure 2).

The gene *DDM1* (decreased DNA methylation 1) encodes a protein that is a chromatin remodeller interacting with the DNA methylation in all sequence contexts (CG, CHG, CHH) and is involved in the maintenance of the DNA methylation and silencing of repetitive sequences, including TE [163]. The mutation of this gene can lead to a reduction of DNA methylation by up to 70% [164]. The gene *MET1* is responsible for the DNA methylation in the sequence of CG and other processes [165,166]. When this gene loses its function, there is incorrect gene expression and activation of TE due to the nearly complete absence of methylation of CG [36,167]. The insertion of the wild type of the *DDM1* gene into a *ddm1* mutant, or of *MET1* into a *met1* mutant, does not restore the DNA methylation at all loci where it was previously lost [168]. The inbreeding of *met1* or *ddm1* mutants contributes to obtaining stable epialleles [166,169]. EpiRILs are nearly isogenic, but they are characterised by the presence of differentially methylated regions (DMRs). DMRs are stably inherited and can influence the plant development, morphology and plasticity, growth rate, nutrient uptake or response to biotic and abiotic stresses [24,124,137,162]. DMRs are also used as markers to identify epiQTLs, which also allows correlating specific epigenomic regions with variation in the trait under study [170], including with flowering time, primary root length or tolerance to salinity [124,171]. Through the research of Liégard et al. [172], for example, numerous epiQTLs were found to be associated with resistance to *Plasmodiophora brassicae*, the causal agent of clubroot. Through the analysis of epiRILs in *A. thaliana*, four epiQTLs were identified to increase the plant resistance to *Hyaloperonospora arabidopsidis* (Hpa) without any associated reduction in resistance to other stresses or plant size [173].

EpiRIL studies reveal that epigenetic modifications are inherited from one generation to the next [174]. They also show that post-translational histone modifications are involved in determining target loci for the RdDM recruitment, which allows the formation of stable epialleles and allows us to understand how they are created. Non-methylated loci, for example, are characterised by the presence of high levels of H3K4me3 and H3K18ac. Tri-methylated H3K4 prevents RdDM recruitment, and the acetylation of H3K18 allows access for ROS1 (Repressor of Silencing 1), which does not allow the recovery of DNA methylation via the RdDM pathway [26,175].

EpiRIL studies have contributed to a better understanding of the phenotypic plasticity in inbred plants [25,61], e.g., as a response to drought and nutrient stress. It is an ability passed on to subsequent generations and possibly subject to selection [61,171]. However, some studies show a smaller impact of epigenetic variation on adaptive traits than had been assumed. With epiRIL, a significant correlation was found between the DNA methylation and glucosinolate accumulation. They are well understood and are mainly found in Brassicales. They are classified as defence substances whose metabolism is regulated in response to many environmental factors, including pathogens and herbivores, but also in relation to the availability of light and water [176,177,178]. Glucosinolates support the stress response of plants and the adaptation to changing environmental conditions through the interactions of signalling pathways [179,180,181]. However, a broad meta-analysis showed that the effect of DNA methylation on this adaptive trait is lower compared to standing genetic variation, which will allow for a faster response to selection than epigenetic variation does [170].

Latzel et al. [182], investigating the epiRILs of *A. thaliana*, showed that the occurrence of epigenetic variation can increase the productivity and stability of plant populations and enhance evolutionary potential [61]. Latzel et al. [182] analysed *A. thaliana* populations composed of epiRIL monocultures and populations composed of 2, 3 or 16 different epiRILs. He observed that epigenetically diverse populations produced more biomass than epigenetically homogeneous populations of this plant. A particular increase in biomass, by up to 40%, was seen in the presence of competitors and pathogens. In the analysis of DNA methylation variation in near-isogenic lines of the population of *Arabidopsis*, it was found that epigenetic variation can lead to functional diversity at the same level as the genetic variation [182]. Examples include the epiRIL population variation associated with a variation in flowering time or higher resistance to pathogens [148,162].

## 6. Conclusions

Inbreeding is associated with many genetic and epigenetic changes. Many studies demonstrate that there are differences in DNA methylation patterns between parental inbred lines and F1 hybrids [4,34,115]. The literature data presented in this review show some aspects of epigenetic changes associated with the inbreeding process, with particular emphasis on environment factors influencing the epigenetic variation and the formation of the phenotypic plasticity. EpiRIL, obtained by crossing isogenic parental lines, characterized by different DNA methylation profiles, were of greatest importance in understanding the impact of the environment on epigenetic variability or epiallele formation and inheritance. Comparative studies between epiRILs and recombinant inbred lines (RILs) show that both epigenetic and genetic variability have similar phenotypic potential. It should be noted, however, that such conclusions may not be absolute truth because epiRILs are characterized by a high epigenetic variability but a very low genetic variability, mainly resulting from the TE activity, while the genomes of RILs show a high genetic variability but also epigenetic variation [154]. In most of the natural populations, there is a distinct genetic variation that may or may not interact with the epigenetic variation. Therefore, more research should be conducted to verify the impact of epigenetic mechanisms on shaping phenotypes in natural populations [183].

Epigenetic analyses allowed us to understand the changes that occur during inbreeding and explain the phenomenon of inbreeding depression. These studies also enabled the explanation of changes in gene expression underlying heterosis. Further research and understanding of the regulation and interactions between DNA methylation, histone modification, sRNA and transcription will allow for even wider use of inbred lines in epibreeding in the future. Proper selection of inbred lines to create hybrids, with expected agriculturally important traits, may turn out to be crucial. Epigenetics and epigenomics contribute to understanding the mechanisms of plant phenotypic variability, the analyses involving epialleles, epiQTL, epihybrids and epiRIL and thus may, in the future, result in the design of effective crop improvement strategies.

## Data Availability

Not applicable.
